# A New Light on Photosystem II Maintenance in Oxygenic Photosynthesis

**DOI:** 10.3389/fpls.2019.00975

**Published:** 2019-07-31

**Authors:** Jun Liu, Yan Lu, Wei Hua, Robert L. Last

**Affiliations:** ^1^Department of Functional Genomics and Molecular Biology, Key Laboratory of Biology and Genetic Improvement of Oil Crops, Ministry of Agriculture and Rural Affairs, Oil Crops Research Institute of the Chinese Academy of Agricultural Sciences, Wuhan, China; ^2^Department of Biochemistry and Molecular Biology, Michigan State University, East Lansing, MI, United States; ^3^Department of Biological Sciences, Western Michigan University, Kalamazoo, MI, United States; ^4^Department of Plant Biology, Michigan State University, East Lansing, MI, United States

**Keywords:** photosystem II, photosynthesis, non-photochemical quenching, repair, fluctuating light

## Abstract

Life on earth is sustained by oxygenic photosynthesis, a process that converts solar energy, carbon dioxide, and water into chemical energy and biomass. Sunlight is essential for growth and productivity of photosynthetic organisms. However, exposure to an excessive amount of light adversely affects fitness due to photooxidative damage to the photosynthetic machinery, primarily to the reaction center of the oxygen-evolving photosystem II (PSII). Photosynthetic organisms have evolved diverse photoprotective and adaptive strategies to avoid, alleviate, and repair PSII damage caused by high-irradiance or fluctuating light. Rapid and harmless dissipation of excess absorbed light within antenna as heat, which is measured by chlorophyll fluorescence as non-photochemical quenching (NPQ), constitutes one of the most efficient protective strategies. In parallel, an elaborate repair system represents another efficient strategy to maintain PSII reaction centers in active states. This article reviews both the reaction center-based strategy for robust repair of photodamaged PSII and the antenna-based strategy for swift control of PSII light-harvesting (NPQ). We discuss evolutionarily and mechanistically diverse strategies used by photosynthetic organisms to maintain PSII function for growth and productivity under static high-irradiance light or fluctuating light environments. Knowledge of mechanisms underlying PSII maintenance would facilitate bioengineering photosynthesis to enhance agricultural productivity and sustainability to feed a growing world population amidst climate change.

## Introduction

Cyanobacteria, algae, and plants convert sunlight into chemical energy through photosynthesis to provide oxygen and food building blocks that are essential for most life forms on earth. Photosynthesis starts with capture of light by light-harvesting antenna, which drives photosynthetic electron flow through photosynthetic machinery comprising several large protein complexes embedded in the thylakoid membranes of prokaryotic cyanobacteria and eukaryotic chloroplasts. Oxygen-evolving photosystem II (PSII) is a highly conserved multi-subunit pigment-containing membrane complex that functions as a light-driven water:plastoquinone oxidoreductase during photosynthetic electron transport (reviewed in Kern and Renger, 2007; Koochak et al., 2019). The electrons extracted from water are converted and stored into organic molecules. Counter-intuitively, PSII is extremely vulnerable to light irradiance, which causes photodamage to PSII reaction centers (reviewed in Townsend et al., 2018; Leister, 2019). The damage is exacerbated if light energy exceeds what can be utilized for carbon fixation, particularly when photosynthetic organisms are subjected to environmental stresses, such as high light, extreme temperature, drought and nutrient depletion, or combined stresses (Ghotbi-Ravandi et al., 2014; reviewed in Murata et al., 2007; Sainz et al., 2010; Salomon et al., 2013; Strzepek et al., 2019; Wilson and Ruban, 2019). The excess light energy also leads to massive generation of reactive oxygen species (ROS) photoproducts, which damage PSII or suppress the repair of damaged PSII (Mishra and Ghanotakis, 1994; Miyao et al., 1995; Okada et al., 1996; Nishiyama et al., 2001; Kale et al., 2017; reviewed in Pinnola and Bassi, 2018). Paradoxically, ROS also act as critical signal molecules to mediate photoacclimation response (Alboresi et al., 2011; reviewed in Wagner et al., 2004; Dogra et al., 2018).

Photoinhibition occurs when PSII suffers from excess light-induced damage or PSII photochemistry is downregulated, resulting in decreased photosynthetic performance and reduced growth and productivity (Kapri-Pardes et al., 2007; Chen et al., 2019; reviewed in Takahashi and Badger, 2011; Wittenberg et al., 2014; Ting and Owens, 2016; Li et al., 2018). Photosynthetic organisms evolved a suite of photoprotective and adaptive mechanisms to prevent or recover from the deleterious effects of photoinhibitory light. These include fast regulatory mechanisms, for instance, movement of chloroplasts away from high-light intensity, reduction of antenna size, induction of alternative electron transport pathways, and slow regulatory mechanisms, such as operation of both enzymatic and non-enzymatic ROS scavenging systems, and triggering systemic acquired acclimation (reviewed in Jarillo et al., 2001; Frigerio et al., 2007; Okegawa et al., 2010; Erickson et al., 2015). Non-photochemical quenching (NPQ) represents one of the fast regulatory mechanisms that is immediately activated and rapidly inducible upon excess solar energy. It protects against excess absorbed sunlight within the PSII antenna by converting photons into dissipative heat (Niyogi et al., 1998; reviewed in Wobbe et al., 2016). In addition, certain organism-specific protein factors evolved to maintain maximal PSII activity under photoinhibitory light conditions (Chen et al., 2018). The land plant-specific thylakoid membrane proteins MPH1 (MAINTENANCE OF PSII UNDER HIGH LIGHT 1) and HHL1 (HYPERSENSITIVE TO HIGH LIGHT 1) evolved to protect PSII against high-light illumination following the transition from aquatic habitats to terrestrial environments (Jin et al., 2014; Liu and Last, 2015a,b). Despite these multi-faceted photoprotective mechanisms, light-induced damage to PSII still occurs. Photosynthetic organisms employ an efficient repair system to replace damaged subunits within PSII reaction centers and restore PSII function (reviewed in Li et al., 2018). A suite of auxiliary proteins, including kinases, phosphatase(s), proteases, and repair/assembly factors have been documented to promote the repair of damaged PSII core subunits (reviewed in Nickelsen and Rengstl, 2013; Järvi et al., 2015). These auxiliary proteins could also cooperate with each other to facilitate the repair process. For instance, *Arabidopsis thaliana* (a flowering plant model species) LQY1 (LOW QUANTUM YIELD OF PHOTOSYSTEM II 1) protein—interacting with HHL1—regulates repair of damaged core complexes to sustain high PSII efficiency upon exposure to excessive light (Lu, 2011; Lu et al., 2011; Jin et al., 2014). Another example is the recent finding that OHP1 (ONE-HELIX PROTEIN1), OHP2, and HCF244 (HIGH CHLOROPHYLL FLUORESCENCE244) form a transient functional heterotrimeric complex assisting in assembly and/or repair of PSII (Hey and Grimm, 2018; Myouga et al., 2018; Li et al., 2019). These repair and NPQ systems may become especially important and could operate in parallel or synergistically to maintain optimal PSII efficiency under fluctuating light environments because photosynthetic organisms live in—and adapt to—their natural growth conditions where light fluctuates rapidly and unpredictably. This review focuses on antenna- and reaction center-based strategies that coexist in oxygenic organisms to minimize the production of the photosynthetic byproducts ROS, thereby safeguarding PSII under changes in light conditions.

## Prevention: Regulation of Light Capture as a Photoprotective Mechanism Across Photosynthetic Organisms

### Non-photochemical Quenching Regulation of Light-Harvesting Efficiency

Photosynthesis is initiated by the capture and trapping of solar energy by light-harvesting systems in thylakoid membranes of cyanobacteria or chloroplasts. However, absorbed light that exceeds what can be used by photosynthesis causes light-induced damage, primarily to PSII. Therefore, maintenance of optimal photosynthetic performance requires efficient regulation of light harvesting for photoprotection. NPQ safely dissipates excess light energy within the PSII antenna system and is found ubiquitously across oxygenic photosynthetic organisms (reviewed in Niyogi and Truong, 2013).

**Figure 1:**
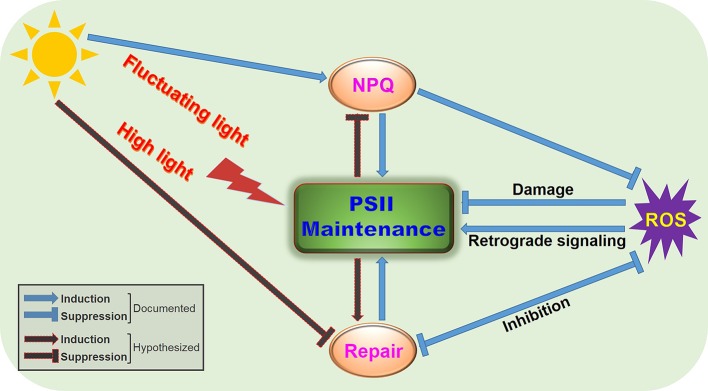
A proposed simplified model illustrating regulation of PSII function by NPQ and repair under fluctuating light environments or high-light irradiance. Fluctuating light or high light can cause damage to PSII and downregulation of PSII photochemistry, with concomitant generation of ROS. To maintain normal PSII function, photosynthetic organisms deploy the antenna-based strategy, NPQ, and the reaction center-based strategy, PSII repair, to efficiently regulate light utilization and energy transfer. ROS act on PSII through damage, inhibition of repair, or retrograde signaling, whose production can be decreased by NPQ or repair. These intricate interplays between NPQ and repair can optimize PSII performance and facilitate acclimation of photosynthetic groups to fluctuating light environments or high-light irradiance.

NPQ responds rapidly and prevents ROS formation during photosynthesis (Figure 1). It is a protective strategy for photosynthetic machinery to acclimatize to excess light conditions. NPQ consists of a variety of processes, such as redistribution of antenna between PSII and PSI to balance electron transport (qT type of NPQ, or state transition) (Bellafiore et al., 2005; reviewed in Erickson et al., 2015), deepoxidation of violaxanthin into zeaxanthin in the xanthophyll cycle and global structural reorganization of PSII-LHCII complexes (Niyogi et al., 1998; Ruban et al., 2007; Park et al., 2019). The most prominent and fastest component is zeaxanthin-facilitated energy-dependent quenching (qE type quenching or feedback de-excitation) (Li et al., 2000; Tian et al., 2019). Because it operates on a time scale of seconds to minutes, rapid and reversible qE is often referred to as flexible thermal dissipation (Demmig-Adams et al., 2006; reviewed in Niyogi and Truong, 2013). qE formation is strictly dependent on a high ΔpH and the PsbS protein but also requires zeaxanthin synthesis (Niyogi et al., 1998; Avenson et al., 2008; Holzwarth et al., 2009). Another NPQ component (qZ type quenching), which is distinguished from qE, is formed within 10–30 min (Nilkens et al., 2010). The formation of qZ is strictly dependent on zeaxanthin but independent of PsbS (Dall’Osto, 2005). The relaxation of qZ depends on zeaxanthin epoxidation and is linked to the kinetics of the zeaxanthin pool. Photoinhibitory quenching is a zeaxanthin-mediated, but not rapidly reversible NPQ component (qI-type quenching or inflexible/sustained thermal dissipation) (Demmig-Adams et al., 2006; reviewed in Pinnola and Bassi, 2018). The relative contribution of each process to the overall NPQ capacity depends on individual photosynthetic organisms and the changing environmental conditions.

Because photosynthetic organisms live in a broad range of habitats, the intensity and spectra of light experienced by different photosynthetic organisms vary extensively. This is particularly true for aquatic organisms, which are subjected to rapidly changing environmental factors, such as abrupt wave movements or phytoplankton migrations. Therefore, it is not surprising that aquatic photosynthetic organisms display distinct photoprotective strategies. For example, the green alga *Chlamydomonas reinhardtii* (a unicellular model species) and diatom *Phaeodactylum tricornutum* both need the LHCSR (LHC STRESS-RELATED PROTEIN) family protein for NPQ formation. Synthesis of the *Chlamydomonas* LHCSR protein is dramatically induced by high light, and it is responsible for the majority of flexible NPQ (Peers et al., 2009; Girolomoni et al., 2019). Notably, the induction of LHCSR expression under high light intensities is found to be controlled by the blue-light photoreceptor phototropin. This suggests that sensing, dissipation, and utilization of light is a concerted process (Petroutsos et al., 2016). Likewise, the LHCSR family protein LHCX1 in *Phaeodactylum* determines NPQ’s high capacity, which correlates with its strong ability to cope with various light stresses (Bailleul et al., 2010; Gundermann et al., 2019). However, the expression and accumulation of LHCX1 is not further induced by excess light energy like it is with *Chlamydomonas* LHCSR (Petroutsos et al., 2016). This demonstrates that *Phaeodactylum* has constitutive and highly efficient photoprotection. These differences in photoprotective capacity between the two aquatic groups reflect their unique ecological adaptations to the sudden, strong changes in underwater light environments.

NPQ mechanisms in terrestrial plants are diverse and elaborate, as reflected by the remarkable diversity of plant species that are distributed in different geographic locations with potentially differential ecological effects. The PsbS (PHOTOSYSTEM II SUBUNIT S) protein in higher plants plays a similar role to the algal type LHCSR. It senses the pH of the chloroplast thylakoid lumen when there is excess light and induces flexible NPQ (Niyogi et al., 1998; Li et al., 2004; Liguori et al., 2019). Short-lived, fast-growing plants such as annual crops have lower qE capacity than long-lived, slow-growing species such as tropical evergreens (Demmig-Adams et al., 2006). It is possible that slow-growing species utilize a smaller proportion of solar energy for photosynthesis, thus having lower intrinsic photosynthetic capacities. In contrast, in overwintering evergreen plants, qI is the predominant NPQ component and it operates independent of PsbS and trans-thylakoid pH, which evolved to cope with combined environmental stresses. The component responsible for qI is correlated initially with sustained D1 protein phosphorylation and xanthophyll cycle arrest and subsequently with newly synthesized zeaxanthin and lutein (Demmig-Adams et al., 2006). This sustained NPQ has critical ecophysiological significance of conferring unique and highly efficient photoprotection in repeated unfavorable seasons over the lifetime of evergreens. It enables these species to downregulate photosynthetic efficiency while continuously harvesting light that does not need to be immediately rechanneled for photosynthesis and growth (reviewed in Demmig-Adams and Adams, 2006). To distinguish the slowly reversible, photoprotective NPQ from qI, this sustained NPQ is termed as qH, which recently has been unveiled to occur in the peripheral antenna of PSII at thylakoid membranes (reviewed in Malnoë, 2018; Malnoë et al., 2018). Genetic screening in *Arabidopsis* discovered that the molecular player of qH is the plastid lipocalin, LCNP (Brooks et al., 2013; Malnoë et al., 2018). Intriguingly, LCNP is a soluble protein localized in the thylakoid lumen, whose expression is induced by stresses such as drought or high light (Levesque-Tremblay et al., 2009). These data suggest that the localization of LCNP to thylakoid membranes likely depends on changes in environments (reviewed in Malnoë, 2018). The importance of sustained quenching is to maintain the normal function of thylakoids by allowing photoprotective NPQ in LHCII under stressful conditions (Lacour et al., 2018; Malnoë et al., 2018).

### Coevolution of Flexible Non-photochemical Quenching and Antenna in Photosynthetic Lineages

The wide distribution of NPQ across photosynthetic prokaryotes and eukaryotes highlights its crucial role in PSII photoprotection. Notably, different NPQ systems have evolved in these diverse photosynthetic organisms. Flexible NPQ (qE), the major and also best-studied component of photoprotective excess energy dissipation, constitutes three systems, which are classified based on their associations with the diversification of the light-harvesting equipment in photosynthetic organisms: the OCP (ORANGE CAROTENOID PROTEIN)-dependent system in cyanobacteria, the LHCSR-dependent system in algae and mosses, and the PsbS-dependent system in mosses and vascular plants (Li et al., 2000; Gerotto et al., 2012; Kosuge et al., 2018; Girolomoni et al., 2019; reviewed in Wilson et al., 2006; Rochaix and Bassi, 2019). Therefore, distinct NPQ regulatory mechanisms have evolved to adjust to differential demands of light energy absorption and utilization, allowing ecological adaptations to specific environments. Intriguingly, these diverse types of NPQ are relevant to the diversified antenna systems during evolution of oxygenic photosynthesis. Cyanobacteria deploy thylakoid membrane-bound phycobilisomes as their light-harvesting antenna (reviewed in Kirilovsky and Kerfeld, 2016) and a special carotenoid molecule within OCP to absorb blue-green light and quench excessive excitation energy from phycobilisomes (Wilson et al., 2006; Mezzetti et al., 2019). Cyanobacterial OCP is both the sensor and site of flexible NPQ (Sedoud et al., 2014; Slonimskiy et al., 2019). Algae and plants utilize transmembrane three-helix LHC antennas, which further diversified into algae- and moss-specific LHCSR proteins. Unlike LHC antennas, LHCSR proteins do not absorb light energy but rather act as quenchers by sensing pH across thylakoid membranes and triggering excess light energy dissipation (Bonente et al., 2011; Pinnola et al., 2013; Tian et al., 2019). In an independent evolutionary innovation from the LHC superfamily, the four-helix protein PsbS in vascular plants functions specifically as a thylakoid membrane pH sensor to trigger and accelerate the formation of NPQ within the LHC antenna (Li et al., 2000; reviewed in Niyogi and Truong, 2013). In contrast to LHCSR (Bonente et al., 2011; Liguori et al., 2019), PsbS neither binds pigments nor quenches excess excitation energy (Bonente et al., 2008; Ruban et al., 2009; Wilk et al., 2013). Therefore, the sensor (PsbS) and the site (LHC) of NPQ are separated in higher plants, which allow high plasticity and flexibility in efficient NPQ induction and recovery.

It should be mentioned that algae also contains PsbS but only accumulates transiently during high light stress, contrasting with LHCSR that accumulates over a much longer period. PsbS shows the ability to increase NPQ but no clear photoprotection activity (Tibiletti et al., 2016). PsbS is unable to compensate for the function of LHCSR in the *lhcsr* mutant (Correa-Galvis et al., 2016). LHCSR alone can explain almost all fast induced NPQ in high light acclimated *Chlamydomonas* cells (Peers et al., 2009). Moss represents a transitional state between algae and vascular plants and has both PSBS and LHCSR. PSBS- and LHCSR-dependent NPQ operate independently and additively (Alboresi et al., 2010; Gerotto et al., 2012). An increased need for flexible NPQ might explain why both LHCSR- and PSBS-dependent NPQ systems are present in early land plants like mosses (Gerotto et al., 2011).

### Exploiting Natural Non-photochemical Quenching Variation to Optimize Photoprotection and Photochemical Efficiency

Natural variation in NPQ capacity is commonly observed in oxygenic photosynthetic organisms, from cyanobacteria to flowering plants, and even between different populations or accessions of the same species grown in the same conditions (Demmig-Adams, 1998; Demmig-Adams et al., 2006; Wang et al., 2017; Hamdani et al., 2019). For instance, different *Arabidopsis thaliana* ecotypes exhibit diverse maximum levels of NPQ: Col-0 and Ws possess lower NPQ compared to Ll-1, Sf-2 (Jung and Niyogi, 2009). The variations in NPQ are not attributable to differences in PsbS or carotenoids required for NPQ formation but to previously unknown polygenic nuclear traits (Jung and Niyogi, 2009). Identification of these genes and understanding the physiological mechanisms responsible for the high NPQ phenotypes should provide a more complete picture of various NPQ systems and potentially lead to approaches for engineering or breeding plants with enhanced photoprotection capability against adverse environmental conditions while maintaining optimal photosynthetic efficiency.

## Operation of Efficient Photosystem II Repair Cycle Allows High Photosynthetic Capacity

Susceptibility to light-induced photodamage and/or photoinhibition, which can be measured as an increase in NPQ component qI, is an intrinsic and unavoidable feature of all PSII reaction centers—from cyanobacteria to flowering plants. The main site of photodamage in PSII is the reaction center D1 subunit, which constantly undergoes rapid turnover (degradation and synthesis) (Aro et al., 1993; Reviewed in Järvi et al., 2015). Although cyanobacteria, algae, and plants have repair mechanisms that differ in detail, they share a central feature: the replacement of the photodamaged D1 subunit with a newly synthesized copy (Armbruster et al., 2010; Kato et al., 2012; reviewed in Nixon et al., 2005; Komenda et al., 2012; Lu, 2016). The PSII repair cycle involves disassembly, targeted reaction-center protein proteolysis, replacement of damaged core proteins, and reassembly of new functional PSII supercomplexes (Haußühl et al., 2001; Kato et al., 2018; reviewed in Nickelsen and Rengstl, 2013). In addition, individual steps in the PSII repair cycle are vulnerable to environmental changes (reviewed in Nath et al., 2013), further necessitating an efficient and timely operation of the repair machinery (Figure 1).

Cyanobacteria and chloroplasts employ distinct PSII repair mechanisms, which may be relevant to evolutionarily distinct thylakoid structures. The photosynthetic membrane systems in oxygenic photosynthetic organisms have evolved into discrete morphological architectures, despite their common ancestry—eukaryotic chloroplasts evolved from cyanobacteria *via* an ancient endosymbiotic event (reviewed in Ku et al., 2015; Bock, 2017). In plant chloroplasts, photosynthetic membranes are differentiated into a network of extensively stacked grana thylakoids and unstacked stromal lamellae. Grana thylakoids are enriched in functional PSII supercomplexes, while the interconnecting stromal lamellae are enriched in PSI and ATP synthase complexes, with cytochrome *b*_6_ *f* complex evenly distributed between the two (Dekker and Boekema, 2005; Daum et al., 2010). In contrast, cyanobacterial thylakoid membranes are not differentiated in grana and stromal lamellae; therefore, their photosynthetic apparatus are not laterally separated (Liberton et al., 2013; Rast et al., 2019).

In higher plants, the individual repair steps take place in discrete subcompartments and occur in a well-defined order (reviewed in Kosuge et al., 2018). Kinases, phosphatases, proteases, ribosomes, and repair/assembly factors are spatially segregated to ensure an operation with minimal interference (Puthiyaveetil et al., 2014; Koochak et al., 2019). Phosphorylation remodels the thylakoid structure to facilitate monomerization of photodamaged PSII supercomplexes in the grana core. These damaged monomeric PSII complexes are then trafficked to granal margins, where dephosphorylation and disassembly likely occur. This allows damaged D1 to be degraded successively by FtSH and Deg proteases (Haußühl et al., 2001; Kato et al., 2012; Krynická et al., 2015; reviewed in Silva et al., 2003; Sun et al., 2007; Tikkanen et al., 2008; Li et al., 2018). The site of *de novo* D1 protein synthesis is located in unstacked stroma lamellae, whereas reformation of active PSII supercomplexes takes place in the highly stacked grana core (Okada et al., 1996; Danielsson et al., 2006).

The green alga *Chlamydomonas* has a thylakoid membrane organization similar to that in higher plants, though with less stacking of thylakoid membranes in its single cup-shaped chloroplast (Wei et al., 2014). Consistent with the less stacking of thylakoids, experimental evidence indicates that individual PSII repair steps in *Chlamydomonas* are not restricted to thylakoid subdomains but rather are dispersed all over in the thylakoids (Uniacke and Zerges, 2007).

PSII repair in cyanobacteria seems to be restricted to specific sites in the thylakoid membranes named repair zones (Silva et al., 2003; Klinkert et al., 2004). Some other studies demonstrated that these repair zones could also be located in the plasma membrane where repair zones converge with PSII biogenesis centers at PDM (PRATA-DEFINED MEMBRANES) subcompartments to allow damaged D1 to be promptly replaced (Schottkowski et al., 2009; Stengel et al., 2012). Another special feature in cyanobacteria is that the conserved phosphorylatable threonine residues in PSII reaction center proteins are not phosphorylated during PSII repair (Calzadilla et al., 2019; reviewed in Komenda et al., 2012). This suggests that phosphorylation- and dephosphorylation-facilitated PSII repair may be a specific step evolved in photosynthetic eukaryotes.

## Appropriate Photosystem II Maintenance Ensures Optimal Photosynthetic Performance Under Natural Fluctuating Light Environments

Photosynthetic organisms experience abrupt and strong changes in light irradiance from seconds to seasons in their aquatic or terrestrial habitats. A multitude of protective and regulatory mechanisms evolved to facilitate their adaptation to such environmental fluctuations. NPQ appears to be a ubiquitous and major light acclimation mechanism that contributes to fitness under varying environments. LHCSR deficiency caused an increased death rate in *Chlamydomonas* following a shift from low to high light, suggesting that LHCSR-induced NPQ is required for optimal survival under variable light conditions (Peers et al., 2009; Kosuge et al., 2018; Girolomoni et al., 2019; Tian et al., 2019). In *Phaeodactylum*, a decreased LHCX1 level led to reduced fitness under stressful light, and even non-stressful light conditions, suggesting that LHCX-dependent NPQ endows diatoms with maximal survival capacity under a wide range of light environments (Bailleul et al., 2010; Gundermann et al., 2019; Park et al., 2019). In *Arabidopsis* plants, NPQ plays a crucial role in rapidly adjusting PSII to artificial fluctuating light (Armbruster et al., 2014, 2016; Duan et al., 2016; Herdean et al., 2016). In field conditions with natural fluctuating light, the NPQ-defective mutants *npq1* and *npq4* exhibited lower PSII activity and produced fewer seeds than the wild type, although they had no visible vegetative growth defects (reviewed in Külheim et al., 2002; Frenkel et al., 2007; Wobbe et al., 2016). Compared to what we know of NPQ in algae and plants, little is known about the importance of OCP-dependent NPQ in cyanobacteria under fluctuating light.

So far, there is no published experimental evidence addressing whether PSII deficiency affects cyanobacteria or *Chlamydomonas* growth under variable light conditions, but several studies in *Arabidopsis* identified protein factors required to safeguard PSII under rapidly changing light conditions. TLP18.3 (THYLAKOID LUMEN PROTEIN 18.3) protein is reported to have a crucial role in adjusting *Arabidopsis* photosynthesis to fluctuating light (Sirpio et al., 2007; Jarvi et al., 2016). The *tlp18.3* mutants did not show visible phenotype under standard growth conditions. However, they exhibited retarded growth under fluctuating light and were highly susceptible to high-light stress. More importantly, the phenotypic defects of the *tlp18.3* mutants were found to be associated with inefficient operation of the PSII repair cycle (Sirpio et al., 2007). Two recent studies uncovered that the loss of *Arabidopsis* PSB27 (PHOTOSYSTEM II SUBUNIT 27) and MET1 (MESOPHYLL-ENRICHED THYLAKOID PROTEIN 1) caused stunted phenotypes when exposed to fluctuating light intensities (Bhuiyan et al., 2015; Hou et al., 2015). These loss-of-function mutations did not affect growth and development under normal light conditions. The reduced vegetative growth in the *psb27* mutant under fluctuating light was attributed to decreased PSII efficiency; this, however, was independent to the PSII supercomplex formation (Hou et al., 2015). The growth retardation in *met1* was due to a defect in the regeneration of active PSII supercomplexes that correlated with the reduced PSII activity (Bhuiyan et al., 2015). Other PSII repair-impaired mutants, including the newly characterized *mph2* and *curt1*, displayed growth retardation under fluctuating light (Liu and Last, 2017; Pribil et al., 2018). The association of decreased growth with impairments in PSII repair suggests that proper maintenance of PSII photochemical efficiency represents an important strategy to ensure plant fitness under adverse light conditions. Exploring the mechanisms of PSII repair in algae and cyanobacteria under fluctuating light may offer further insight into the evolution of photosynthesis. Moreover, exploiting PSII repair mechanisms could be promising targets for bioengineering photosynthesis to increase photosynthetic capacity and productivity under controlled photoinhibitory light and natural fluctuating light environments.

## Optimizing Non-Photochemical Quenching to Enhance Photosynthetic Capacity and Growth in Field Conditions

Deeper understanding of NPQ mechanisms should inform strategies to optimize the balance between photoprotection and photosynthetic productivity. Optimization of photoprotection to improve photosynthetic performance is an emerging strategy in agriculture. It is generally accepted that the solar energy conversion efficiency for crop plants is much lower than the theoretical maximum yield (~12%) (reviewed in Walker, 2009; Blankenship et al., 2011). One major cause for the low efficiency is that upper leaves of a canopy absorb more sunlight than can be used for photochemistry, while photosynthesis of lower leaves is limited by shading (reviewed in Long et al., 2015). Altering the pigment content and leaf arrangement in the canopy may improve crop yield. A smart canopy with even light absorption would have light green vertical leaves at the top of the canopy and dark green horizontal leaves at the bottom (reviewed in Ort et al., 2015). Therefore, an optimized canopy may achieve higher crop yield.

Another major reason for the lower than the expected maximal photosynthetic efficiency in crops (and other plants) is that NPQ relaxation lags behind fluctuations in sunlight during sudden transitions from high to low light. This happens when passing clouds or movement of neighboring leaves/plant species shade sunlit leaves. The slow NPQ response could cost up to 30% of carbon gain (Zhu et al., 2004, reviewed in Zhu et al., 2008), suggesting that accelerating NPQ relaxation would be a strategy for increasing photosynthetic productivity. For example, speeding up the response to natural shading events by enhancing the recovery from photoprotective NPQ in *Nicotiana tabaccum* markedly increased photosynthetic capacity and bulked up leaves, stems, and roots, which contributed to a 15% gain in plant biomass production in field conditions (Kromdijk et al., 2016). Much more rapid NPQ induction in bright light and much faster NPQ relaxation following a drop in light intensity enable plants to track fluctuations in sunlight more closely, contributing to more efficient light energy utilization and carbon fixation. This proof-of-concept field trial opens the door to enhancing photosynthetic performance and productivity in agricultural and natural ecosystems.

## Concluding Remarks and Future Perspectives

In oxygenic photosynthesis, it is important to (1) safely handle excess absorbed light energy that would otherwise cause massive ROS production and damage the photosynthetic machinery and (2) efficiently convert solar energy into chemical bond energy. Tight regulation of these two aspects may contribute to an increase in productivity in agriculture and natural ecosystems. Understanding the elaborate NPQ mechanisms and the robust PSII repair systems may help identify targets to optimize photosynthetic efficiency. This would facilitate translational work toward exploring yield potential to sustainably meet the global rising demands for food, fuel, and fiber in the future climate change. Prior to accomplishing these grand goals, multiple outstanding questions await to be addressed:

1. Do antenna-based photoprotection and reaction center-based repair operate in concert or in parallel to regulate PSII efficiency and photosynthetic capacity under photoinhibitory light and other environmental stresses? How does evolution of NPQ in the oxygenic organisms contribute to that of repair and vice versa?
2. How do ROS regulate PSII activity under fluctuating light environments or field conditions?
3. Are the molecular mechanisms of PSII repair under changing light different or similar to those under high-light irradiance? Can photosynthetic species discern PSII damage caused by these two types of light conditions and initiate distinct repair strategies?

## Author Contributions

JL and RL conceived the project. JL, YL, WH, and RL wrote and edited the manuscript.

### Conflict of Interest Statement

The authors declare that the research was conducted in the absence of any commercial or financial relationships that could be construed as a potential conflict of interest.
